# Exploring the promises of intersectionality for advancing women's health research

**DOI:** 10.1186/1475-9276-9-5

**Published:** 2010-02-11

**Authors:** Olena Hankivsky, Colleen Reid, Renee Cormier, Colleen Varcoe, Natalie Clark, Cecilia Benoit, Shari Brotman

**Affiliations:** 1 Public Policy Program, Simon Fraser University Vancouver - Harbour Centre Campus, 515 West Hastings Street, Vancouver, BC V6B 5K3, Canada; 2 Institute for Critical Studies in Gender and Health, Simon Fraser University Vancouver - Harbour Centre Campus, 515 West Hastings Street, Vancouver, BC V6B 5K3, Canada; 3 Women's Health Research Network, Simon Fraser University - Harbour Centre Campus Room 3277, 515 West Hastings Street, Vancouver, BC V6B 5K3, Canada; 4 School of Nursing, University of British Columbia, T201 UBC School of Nursing, 2211 Wesbrook Mall, Vancouver, BC V6T 2B5, Canada; 5 School of Social Work and Human Service, 900 McGill Road, PO Box 3010, Kamloops, BC V2C 5N3, Canada; 6 Department of Sociology, University of Victoria, P.O. Box 3050 STN CSC, Victoria, BC V8W 3P5, Canada; 7 School of Social Work, McGill University, Room 300, Wilson Hall, 3506 University Street, Montreal, Quebec H3A 2A7, Canada

## Abstract

Women's health research strives to make change. It seeks to produce knowledge that promotes action on the variety of factors that affect women's lives and their health. As part of this general movement, important strides have been made to raise awareness of the health effects of sex and gender. The resultant base of knowledge has been used to inform health research, policy, and practice. Increasingly, however, the need to pay better attention to the inequities among women that are caused by racism, colonialism, ethnocentrism, heterosexism, and able-bodism, is confronting feminist health researchers and activists. Researchers are seeking new conceptual frameworks that can transform the design of research to produce knowledge that captures how systems of discrimination or subordination overlap and "articulate" with one another. An emerging paradigm for women's health research is intersectionality. Intersectionality places an explicit focus on differences among groups and seeks to illuminate various interacting social factors that affect human lives, including social locations, health status, and quality of life. This paper will draw on recently emerging intersectionality research in the Canadian women's health context in order to explore the promises and practical challenges of the processes involved in applying an intersectionality paradigm. We begin with a brief overview of why the need for an intersectionality approach has emerged within the context of women's health research and introduce current thinking about how intersectionality can inform and transform health research more broadly. We then highlight novel Canadian research that is grappling with the challenges in addressing issues of difference and diversity. In the analysis of these examples, we focus on a largely uninvestigated aspect of intersectionality research - the challenges involved in the process of initiating and developing such projects and, in particular, the meaning and significance of social locations for researchers and participants who utilize an intersectionality approach. The examples highlighted in the paper represent important shifts in the health field, demonstrating the potential of intersectionality for examining the social context of women's lives, as well as developing methods which elucidate power, create new knowledge, and have the potential to inform appropriate action to bring about positive social change.

## Background

Like most social movements, the women's health movement has given voice to those who are often marginalized by society and given limited, if any, decision-making power in setting health policies and priorities[1]. The efforts of women's health activists in Canada over the past 20 years has resulted in key events, developments, and advancements that have earned Canada the reputation as a world leader in women's health research [2-6]. This reputation, however, should be questioned. One central weakness has been that, despite its efforts to be inclusive, the traditional foci of Canadian health research on women tends to essentialize the category of women (that is, assumes that all women, regardless of age, cultural background, geographical location, socio-economic status, religion, sexual orientation and other categories of difference, share exactly the same experiences, views, and priorities), and further, gives too much primacy to gender over other key determinants and does not adequately address the interactions among all determinants of health[7]. Consequently, the issues and priorities of many vulnerable women, including members of ethnic, racial, and linguistic minorities, Aboriginal women, low-income women, lesbians, and women with disabilities are usually excluded from mainstream women's health research[8].

For example, in considering the attention to Aboriginal women's health in Canada, Dion Stout, Kipling, and Stout[9] argue that "most of the work being undertaken remains narrowly focused and is often tangential to the underlying causes of Aboriginal women's marginalization and oppression" (p. 17). There is also a lack of appropriate research on immigrant and refugee women from racialized communities. Among other things, resilience among these groups of women tends to be overlooked and they are not seen as resources for their own health care needs and priorities[10,11]. A similar situation exists for lesbian, bisexual, and transgendered individuals who remain largely understudied by women's health researchers[12]. Bernard[13] correctly notes that women's health researchers today need to ask " [w]hat communities do they serve?". Indeed, the question of which women are benefiting and which are excluded from or are marginalized in current research and policy agendas and priorities needs to be considered.

In response to these pressing issues, and combined with the increasing diversity within Canada (e.g., Canada is home to people of more than 200 different ethnic origins; Statistics Canada, 2008), more and more calls are being made to be attentive *explicitly *to the diversity among women[2]. In her review of gender based analysis in research and policy, Hankivsky[14] calls for "an altogether new conceptual framework that combines intersecting axes of discrimination but does not privilege gender over other determinants of health" (p. 159). And in their examination of women's health in Canada, Varcoe, Hankivsky, and Morrow[15] call for a critical reflection on the concept of women's health and the issues that arise from complex understandings of gender and its interactions with other forms of social difference. However, "recognizing the diversity of women poses special problems for women's health because it challenges the singular political strategy used for generating change, that is the foregrounding of inequities based on gender"[16] (p. 16).

To illustrate, in Canada there are various frameworks that promote a sex and gender analysis in health research (i.e., gender-based analysis, sex and gender-based analysis, gender and sex-based analysis) and the assumption often made is that these are effective universal models for capturing issues of diversity because, as argued by Weldon[17], "gender analysis must incorporate analysis of race, class, sexuality, and other axes of disadvantage, and explore interactions among them" (p. 236). Although some researchers who rely on a gender and/or sex based analysis do incorporate other variables of investigation, many, due to habit, ease of analysis, and/or ideological or political agendas, continue to limit their analysis to comparisons of women and men, producing binary data[18]. Alternatively, they assign prominence to gender or sex and use an 'additive' approach which entails looking at numerous variables as separate and dichotomous rather than mutually interdependent and interactive[19].

Additionally, these types of research practices often fail to recognize or address issues of power. For instance, women's health research and policy seeks to find commonalities of experience while intending to represent a broad constituency. And yet research processes such as the establishing of priorities, areas of investigation, and approaches to research, are typically controlled by a privileged group of individuals (both men and women), who for the most part, do not recognize their privilege or are not even conscious of "the performance they enact, perceiving them to be 'just what is normal"'[13] (p. 360). Moreover, many researchers do not represent marginalized positions or voices and often design and produce research that tends not to benefit anyone who differs from the privileged white 'norm.' This dynamic is described in the context of psychology by Cole[20] who argues:

Because political agendas are often set by subpopulations within a constituency that have relatively more privilege and therefore more status and sometimes, resources, other subgroups may experience secondary marginalization in which their interests are not addressed by the organizations or movements purporting to serve them (p. 445).

In response to such issues and due to the lack of progress in reducing health inequities more generally, a growing number of women's health researchers and advocates are calling for and engaging with research that explores multiple axes of difference - - namely, intersectionality-type research [15,21-24].

## Intersectionality

Intersectionality has a long history but was popularized by the work of Crenshaw[25] and is now recognized as a research paradigm [19,26,27] that is based on a number of key assumptions concerned with the simultaneous nature of multiple categories at multiple levels. First, is the idea that "different dimensions of social life cannot be separated into discrete or pure strands"[28] (p. 76). Related to this is the notion that no one category of social identity is necessarily more important than any other. Staunæs[29] explains: " [i]n principle, there is not a predetermined or pre-hierarchical pattern between categories. It is not gender first, then ethnicity, or the reverse, first ethnicity, then gender" (p. 105). Intersectionality analysis resists essentializing any categories (i.e., treating all members of a single social group as the same and assuming they share the same experiences), being attentive to time, place, and historical and localized specificity. As Yuval-Davis[30] argues, "social divisions are constructed by, and intermeshed with each other in specific historical conditions" (p. 200).

Second, an intersectionality analysis does not seek to simply add categories to one another (e.g. gender, race, class, sexuality), [31,32] but instead strives to understand what is created and experienced at the intersection of two or more axes of oppression. In so doing, it recognizes the multidimensional and relational nature of social locations and places lived experiences, social forces, and overlapping systems of discrimination and subordination at the centre of analysis[33]. In this way, an intersectionality analysis captures several levels of difference. In particular, it reveals how intersecting forms of discrimination and oppression create opportunities and social and material benefits for those "who enjoy normative or non-marginalized statuses such as Whiteness, maleness, heterosexuality, or upper class status"[34] (p. 808).

Third, it is generally understood that those who engage in intersectionality research or policy are committed to social justice and seek significant shifts in power. Because intersectionality recognizes relational constructs of social inequality, it is an effective tool for examining how power and power relations are maintained and reproduced[29]. Weber and Parra-Medina[35] elaborate: "intersectional models assume a connection between oppression and resistance, between gaining knowledge of oppressive systems and engagement in social activism to challenge them" (p. 188). Intersectionality scholars recognize the need to work with a variety of stakeholders (e.g., policy makers, grassroots activists and community groups, including multiply oppressed communities) to undertake research and policy, and to make social change.

## Intersectionality Methodology

In general, intersectionality scholarship, and specifically methods for developing research studies from an intersectionality perspective, remain at the margins of health research and policy[19,36,37]. Hancock[38] has argued that " [o]ne area of research that remains under- explored within intersectionality is the development of research designs and methods that can capture effectively all of the tenets of intersectionality theory" (p. 79). Most recently, Nash[39] concluded there is a "lack of clearly defined intersectional methodology" (p. 4). The development of intersectionality methodology holds the promise of opening new intellectual spaces for knowledge and research production[40] and has the potential to lead to both theoretical and methodological innovation[27] in women's health research and in policy and policy analysis innovation. What is most problematic for scholars undertaking women's health research from an intersectionality perspective is under-developed research methods. Even self-identified intersectionality scholars are themselves struggling with how to operationalize intersecting categories when initiating and developing research projects. And yet, as Iyer and colleagues[41] have recently argued, " [i]nsufficient attention to intersectionality in much of the health literature has significant human costs," (p. 13) including in terms of morbidity and mortality costs associated with health disparities.

For a number of reasons, translating intersectionality theory into methodological practice is not easy[42]. First, there is a disconnect between intersectionality scholarship and the conceptualization of research questions and designs[26]. Second, there is a lack of certainty as to how, when, and where intersectionality frameworks should and can be applied[43]. Researchers often find it difficult to interrogate their 'blind spots' and are not clear about how to (re)consider the topics of their research and their research designs in light of the variety and density of multiple differences[7,18,44-46]. Third, the difficulty of applying intersectionality to empirical designs, especially in areas dominated by quantitative research, has also been highlighted[47-49]. Fourth, little work has been done to determine whether all possible intersections might be relevant at all times, or when some of them might be most salient [49,50]. Fifth, intersectionality requires access to pertinent health information that often does not exist - - for example, data that represent multiple groups and which reflect significant variations within those groups across genders, socioeconomic statuses, social classes, and sexual orientations[40].

What is required at this time is ongoing debate and deliberation for moving intersectionality scholarship forward and explicit attention as to how intersectionality can inform research design, evidence production, and knowledge translation[45,50]. Further, researchers who seek to use an intersectionality approach need to confront how to "tolerate and engage with the encounter with an/other, without assimilating that other to received structures and so robbing them of that difference or otherness"[51](p. 304). Applebaum[52] observes that many researchers, who seek to understand the "Other" engage in "a certain form of voyeurism and exploitation that further reinscribes privilege and marginalization" (p. 363) instead of beginning any research process with taking into account where they are located in the hierarchies that structure social order[46,53]. Researchers therefore need to think about their intentions in undertaking intersectionality research, what key assumptions they bring to the research[27], how to engage in "reflexive, critical and accountable feminist inquiry"[44] (p. 79), and the "different possibilities of interacting and positioning and establishing certain subject positions"[29] (104) in terms of the entire research process.

## Canadian Examples of Intersectionality Research

McCall [54] defines intersectionality methodology as "...a coherent set of ideas about the philosophy, methods, and data that underlie the research process and the production of knowledge" (p. 1774). In keeping with this broad conceptualization of intersectionality methodology, examples of Canadian health researchers explicitly adopting intersectionality as a theoretical concept and tool for research are beginning to emerge[22,55-71]. In the following sections, five examples of Canadian researchers who identify themselves as women's health and/or gender and health researchers and who explicitly use an intersectionality analysis in their work are highlighted.* Each provides an example from one of their health research projects describing how their work was informed by an intersectionality perspective, how intersectionality was incorporated into their work, and the challenges they faced in conducting intersectionality research. In particular, all the authors were asked to attend to the following questions about their research:

1) Who is being studied? Who is being compared to whom? Why?

2) What issues of domination and exploitation are being addressed by the research? Is the issue power at the centre of all analyses?

3) How will human commonalities and differences be recognized without resorting to essentialism, false universalism, or obliviousness to historical and contemporary patterns of inequality?[20]

4) How do researchers make sure that they are not seeing what they want to see in their research?[53]

5) In order to affect social change, does the research include representation from all key stakeholder groups such as policy makers, grassroots activists and community groups, including multiply oppressed communities?

6) Is the research framed within the current cultural, societal, and/or situational context? (Adapted from Hankivsky and Cormier[19])

Moreover, to ensure a level of consistency and cohesion in the presentation of these diverse case studies, each author was asked to highlight the following information about their studies: the research question and/or goal and objectives, the type of study that was conducted, data collection methods and analysis, conclusions about their research and finally, the challenges of undertaking intersectionality research as well as how an intersectionality analysis benefitted their study.

### Varcoe and Dick: The Intersecting Risks of Violence and HIV for Rural Aboriginal Women in a Neo-Colonial Canadian Context

Colleen Varcoe, from the School of Nursing at the University of British Columbia and Sheila Dick, a Counsellor/Family Support Worker from the Canim Lake Band, Tsq'escenemc Nation led a study [68-70] involving 30 rural women who reported being victims of partner violence and who believed they may have been at risk for HIV. Forty-two community representatives, including social service providers, and leaders from Aboriginal and non-Aboriginal communities and organizations also took part in the study. Participants were recruited using community based approaches such as advertisements in local newspapers and through word of mouth. Using a critical ethnographic approach, women were interviewed individually at a safe place of their choosing. The women were offered a choice of interviewer so that they could be interviewed by someone from their own community known to them (either a local Aboriginal or non-Aboriginal woman), or an academic of mixed heritage. Interviews lasted an hour and half on average, were tape recorded, and fully transcribed. The women were offered an honorarium of $30 to recognize their time and contribution. Six of these women also participated in a subsequent focus group after having read a preliminary analysis of individual interviews. The community representatives--four men and 38 women--read and discussed the analysis of the women's experiences in the six focus groups. Using an intersectionality framework, the experiences of Aboriginal and non-Aboriginal women were compared to illustrate both similarities among all women, regardless of their ethnicity, with regards to how poverty, gender, and geography shaped their experiences and the ways in which colonization and racism affected them differently.

The research was framed within the current cultural, societal, and economic context of rural and Aboriginal communities in British Columbia and was shaped through the broader context of global economics and neo-colonialism. Gender and power relations, both within interpersonal relations and within the context of globalization, were central to understanding women's risks of both violence and HIV. Further, gender and power relations intersected with rural geography and economics with particular effects for women. For Aboriginal women, these intersections were deeply shaped by historical power relations of colonization and racism as they play out at structural and individual levels.

In this work, the researchers were challenged with the need to infuse the analysis with an understanding of the historical and contemporary patterns of inequity, sustained by colonization and racism, while continuously resisting sliding into essentialist categories - - particularly Aboriginal versus non-Aboriginal. Even while attempting to foreground the complexity of women's lives, Varcoe and Dick acknowledge that they may have inadvertently added to the tendency to falsely universalize all rural women's experiences, or all Aboriginal women's experiences. In particular, all rural Aboriginal women were not economically impoverished, and were not at high risk of violence. Importantly, the fact that Varcoe and Dick were very differently positioned socially may have assisted them in resisting essentializing and falsely universalizing tendencies and ideological impositions. Varcoe is an academic and an urban-dwelling woman of mixed Caucasian and Aboriginal ancestry with no Aboriginal family ties; Dick is a community worker, a rural-dwelling woman from the Tsq'escenemc Nation who has lived both on and off reserve, and who is a survivor of the residential school system. Together they maintained a critical analytical stance toward their own analysis. For example, Dick helped Varcoe see subtle evidence of her assumption that there had been a non-violent, or pre-violent time of 'normalcy' in the women's lives. However, the positionality of researchers does not necessarily buffer against essentializing the experiences of the populations of interest in research.

Another challenge identified by Varcoe and Dick was how to provide a complex understanding of women's intersecting risks of violence and HIV, and draw attention to how various forms of oppression shape experience, without contributing to stereotypes of Aboriginal women in particular. The strategies employed to mitigate this problem included a) insuring that both Aboriginal and non-Aboriginal women were included in the study (the impetus for the study arose from citizens and service providers concerned about the intersection of HIV and violence and the need to develop an HIV prevention strategy, concern that initially focused on Aboriginal women), b) involving diverse community representatives to guide the study through an advisory committee, c) analysing and presenting data carefully so as not to erroneously connect stigmatizing issues (e.g. substance use, sexually transmitted infections) only with Aboriginal women, and d) seeking out and foregrounding non-stereotypical and non-stigmatizing 'cases' (for example, highly educated Aboriginal women in reasonably well paid jobs). At the same time this challenge required resisting the possibility of simply mining the data for evidence that added to their pre-existing concerns about how racism and colonization shape Aboriginal women's lives.

Grounded within an intersectionality perspective and specifically within a social justice agenda, the research engaged, from its initial conception, a wide range of key stakeholders from the local level grassroots activists and community groups, service providers, and leaders. Of note, however, was that the engagement of all stakeholders over the long term, and in particular, policy makers beyond the local level, was not sustained largely because of existing power dynamics. While some important relationships between individuals were maintained, this development further underscored the need for an intersectionality approach, which specifically addresses issues of power, in the processes of knowledge uptake and knowledge translation.

In sum, this project demonstrated the significance of deconstructing essentializing conceptions of 'Aboriginal' populations in health related research and properly identifying, as an intersectionality perspective calls for, the full range of interlocking factors that affect experiences of health, and in this instance, within rural contexts. This also points of course to the importance of disentangling other often essentialized categories in health research, such as 'lone mothers', 'immigrants,' 'refugees,' 'seniors,' and 'persons with disabilities.'

### Brotman and Colleagues: Exploring the Health Needs of Gay and Lesbian Aging Adults

Shari Brotman and her colleagues used participatory methods to examine intersectional identity and multiple forms of discrimination in health among lesbian, gay, bisexual, and two-spirit communities[57-59,72-75]. They conducted a five year qualitative research study on the health and social service needs of gay and lesbian seniors, their families and their caregivers in Canada through an exploration of their experiences in accessing long-term-care services and in their perceptions of needs as they or their loved ones age. The research also set out to examine professional and institutional interpretations of need and current responses.

Challenges in recruitment required the team to extend data collection to over three years. Recruitment efforts emphasized the importance of respecting the positions/identities of individuals and stressing the confidentiality of interview processes in order to respond to potential participants' concerns. A snowball sampling technique was the primary method of finding participants for the study [76-79], and researchers recruited 38 senior participants (18 men and 20 women).

Interviews lasted approximately 1 1/2 to 2 hours. The interview protocol was semi-structured, with open ended questions in several theme areas that provided participants with space to discuss issues important to them. Analysis was undertaken using the Grounded Theory method outlined by Glaser and Strauss[80] and Strauss and Corbin [81]. The goal of the analysis was to identify themes and the relationships between these themes. An intersectionality framework was explicitly incorporated in to the analysis process. This meant that content analysis of transcripts was guided by an attention to both how identity was experienced and constructed in participants' "talk" and the ways in which such issues as access and experiences of discrimination were shaped by multiple social locations. In order to ensure that the research process and the findings were authentic with respect to the voices and meanings of participants themselves[82], we employed techniques such as member checking.

The articulation of intersectionality in the "doing of research" led Brotman and her colleagues to explicitly identify both the process and outcomes of research as social change oriented. This meant that all aspects of research design, implementation, analysis and knowledge-sharing of results were developed through the collaborative partnership of community members, health care providers, and university-based academics. An intersectionality analysis benefitted the study immensely. Examining the lived experiences of gay and lesbian seniors required an approach which focused on building connections between identity, social location, and multiple forms of discrimination in health. This framework blended an understanding of the more structural elements of social location with the more personal interpretive aspects of what it means to experience aging in diverse and heterogeneous ways. Drawing on diverse social locations was intended to address both the multiple experiences of aging, and counter tendencies to essentialize the experience of growing old as a gay man or lesbian (that is, assuming that the experiences are the same for all members of these groups). Researchers also sought to understand how growing old is experienced as structural, biological, relational, and personal, and that these processes were inherently tied to the concept and practices of power. For example, older gay men and lesbians were often considered to hold a powerless position within society as well as within health and social care encounters. This powerlessness was both real and material. As a result, gay and lesbian seniors experienced marginalization and/or structural or systemic discrimination. These were related to social or legal rights (e.g., sexism, classism, ableism, and homophobia as represented in the current study) as well as minority, marginal or stigmatized positions within society. Experiences were analyzed and presented as being produced in the intersections of multiple social locations within a social context.

Using an intersectionality analysis enabled the research team to explicate processes of identity negotiation, discrimination, and access to care within a framework which understood power as relational. Older gay men and lesbians and their families are not only acted upon, they are also actors with agency who can respond and resist social circumstances, processes, and structures which marginalize and oppress them. This complex articulation of the intersection of identity, social location, and discrimination lent credibility to the study. This, researchers believed, resulted in its uptake by the most important people and communities, namely gay and lesbian seniors themselves and their families. Consequently, knowledge-sharing with respect to the research results, continue to shape many local initiatives, media awareness, workshops, presentations, training, and policy activism across the country.

### Clark and Hunt: Commercial Sexual Exploitation - Innovative Ideas for Working with Children and Youth

Natalie Clark, a researcher based at Thompson River University in British Columbia, and Sarah Hunt, an Aboriginal community-based researcher, integrated an intersectionality perspective with an action research methodological framework in their studies exploring violence in the lives of sexually exploited youth and adults in the sex trade[63,64]. Data were collected by Clark and Hunt, through focus groups and interviews, and in partnership with community based researchers and advisory groups established in each location, and with a provincial advisory committee overseeing the project. Five communities and 110 individuals participated in the research. At the time of the research, Clark and Hunt were both community based researchers, well versed in the principles of feminist participatory action research and community based participatory action research which acknowledge power, social justice, and action outcomes. However these approaches failed to address the complexity of sexual exploitation within these communities in British Columbia (BC). An intersectionality framework was essential in framing the questions within the context of colonization in BC, and in consideration of the diversity of communities within which the research occurred (i.e., urban and rural, on-reserve and urban Aboriginal).

As part of this work, Clark and Hunt developed an "Intersectional Research Team", a term they coined to describe a team which analyzes the role of the researchers within an intersectionality approach and that reflects the diversity and intersections of the issue and the communities that are the focus of the study[83-86]. Too often, research teams consider the application of intersectionality towards the subject of their research but fail to begin with themselves as researchers. In order to move beyond additive or tokenistic approaches, members of an Intersectional Research Team began with an analysis of themselves and each other in this process. Researchers were encouraged to locate themselves with respect to the communities and issues and to consider the experiences of insider, outsider, and the spaces in between. Each researcher began with considering questions that help identify their own social location and the complexity of their identity with respect to the research being undertaken.

Within an Intersectional Research Team there is the acknowledgment of the multiple identities of each of the researchers (see Clark et al. 2009[83] for a fuller description of this and the tool developed by Clark and Hunt). Just as the social locations and identity of the research participants can shift and change during the process of a research project, so too can those of the research team members. Shifting locations requires a self-reflective stance which is contextual and fluid. Through considering and challenging the binaries of insider and outsider, the Intersectional Research Team continually engaged with the multiplicity of the members of the research team and their connections to the people and issues being examined.

For example, Clark's parenting status changed during the several years working of this research project. Clark shifted from a single urban-dwelling white woman to becoming pregnant "out of wedlock." Clark's children include a mixed-race girl and twin boys who are Aboriginal and from the Chase area (one of the research communities). Not only did Clark's parenting status have a direct impact on relationships with the communities that were part of the study, but the reality of raising three children alone in East Vancouver resulted in a return to a rural community. After 17 years in Vancouver, Clark, now a mother of 3, became an academic by joining a rural university, Thompson River University. During this time, Clark's grandmother also died whereupon Clark learned of her grandmother's hidden Aboriginal identity and her own Metis heritage, adding further complexity to the English, Welch/Irish identity she named in locating herself within the various territories and communities she entered.

Conducting focus groups in communities throughout BC while pregnant altered Clark's relationships with the participants since many of the young women were pregnant themselves and also without partners. In becoming an "inappropriate Other" herself, Clark as a researcher shifted her outsider status to having a more direct connection with the young women being interviewed. This allowed the moral dimensions of mothering and the social construction of good mother/bad mother to be in the foreground. Many of the young Aboriginal women spoke about the role of parenting status in their community and culture and how their own identities had shifted as they became parents. One of the young women, and a community-based youth researcher, gave birth to a baby while part of the project and was part of the research presentation at the Justice Institute of BC with her baby - whom she also named "Justice" - - aptly capturing the vision underlying the research. This example of the shifting and fluid nature of identity within one researcher who was part of the team demonstrates the importance of an intersectionality perspective in understanding the research team itself from framing the question, to conducting the research, and analyzing the data. Further, an intersectionality framework and community based action research approach ensured that knowledge translation and action outcomes were defined by each community. Other action outcomes included provincial level recommendations for the justice system and the development of long-term relationships and connections between researchers and community members across the province.

An intersectionality framework allowed for the complexity and diversity of the issue and of the participants to be identified in policy and practice recommendations. In rural areas of BC, there was recognition of the need for capacity building and knowledge with respect to the issue itself; therefore, educational workshops were offered in remote northern communities to address this and to avoid essentializing rural communities and/or the issue of sexual exploitation and violence within these communities. Finally, an intersectionality framework allowed the team to identify the importance of addressing gaps in research, including the inclusion of the experiences of male, and transgendered youth and adults in our understanding of the complexity of violence.

### Benoit and Colleagues: The Stigma Project

The *Stigma Project *sought to advance understandings of the social factors impacting the health, safety, and well-being of sex workers. Specifically, the researchers aimed to shed light on the impact of low prestige and discrimination on a diverse community of sex workers in comparison to two other occupational groups who provide "emotional labour" as a large part of their jobs - hairstylists and food and beverage servers [56,87].** Cecilia Benoit, from the University of Victoria and her colleagues predicted that the intense stigma attached to sex work in Canada would have an independent effect on sex workers' health, safety, and well-being, but that other social factors, including gender, sexual orientation, age, income, education, Aboriginal status, would intersect to result in within-group variation among sex workers, as well as the other two occupational groups.

The Stigma Project approached sex work as a form of economic activity that shares many of the habitual, ordinary qualities of other service work in the formal economy. The team adopted a social determinants of health framework and employed an intersectionality methodology, both of which offered useful tools for studying and analyzing the different social factors operating in society that play a hand in shaping the trajectory of peoples' occupational lives. A social determinants perspective aims to identify not only how these factors individually impact health within a population, but also the reasons why there are differences in health outcomes across the life course; and further, how these differences are shaped by unequal access to health care and other key resources[88,89]. The essential insight of intersectionality is that various dimensions of social stratification--including SES, sex, gender, thnicity, race, age and others-can add up, or cumulate, to great disadvantage for some groups of people[23]. What an intersectionality approach emphasizes is the need for a context-specific approach[90] - one that is careful not to conflate SES, sex, or gender nor ignore the other reported factors determining the health of groups in particular locations at particular points in time[7].

The Stigma Project was designed as a collaborative effort between Benoit and her university-based research team and an advisory group that was instrumental in designing and shaping the project from the beginning to the end. The advisory group was made up of representatives from the three occupations as well as frontline service agencies [56,91,92]. The Stigma Project incorporated the following research techniques: collecting contact telephone numbers for respondents and significant others at the first wave interview; phoning respondents periodically between waves to maintain contact; and establishing a study-specific email address so respondents could contact the study. At each wave, respondents were interviewed and were asked to complete self-report questionnaires. The study followed respondents across the four interview waves even if they exited their line of work and/or changed geographical locations.

Data collection instruments for the Stigma Project combined standardized measures, items used in studies of populations with high health risk (e.g., AIDS research), and measures unique to the research project. Data were also collected on demographic and background information; family history; current physical and mental health status; work conditions, occurrence and time of onset of serious health conditions, and occupational health and safety issues. Benoit and her colleagues were able to follow workers (and their children) from these different occupations over the three-year period and document changes in their working conditions, access to health services and health status through repeated interviews, and examine the impact of overt and covert discrimination on their health and well-being and that of their children.

Results to date from the Stigma Project confirmed the two hypotheses: 1) fundamental factors (gender, education, and income) are linked first, to poorer self-reported health for frontline workers compared to the general population and second, to worst self-reported health for sex workers compared stylists and servers; 2) both working conditions and critical life events were found to intervene between frontline service workers and depression levels, with critical life events and chronic stressors playing a more significant role. The findings also indicate that sex workers were more likely to use some substances (cocaine, crystal meth, heroin, Non-prescribed Rx and Club drugs), while stylists reported the highest rate for alcohol and marijuana use. Thus, all three groups used addictive substances but usage for sex workers was more serious, likely relating to their overall greater structural vulnerability[93]. In addition, while all three occupational groups experienced difficulties getting treated for their main health problems, sex workers faced the greatest difficulties. Reasons for difficulty in accessing services related to the workers' occupation, access to key resources, and perceived discrimination by providers[94]. Finally, experiences of stigma were not uniform but were linked to identities with multiple disadvantages. Social networks provided some supports but were also sources of distress.

Currently, the authors are conducting further analyses to tease out under what contexts the identified factors have greater power in explaining the structural vulnerabilities faced by these different groups of workers and the variation in their health outcomes that cut across essentialist lines. The advisory committee has also helped the researchers to guard against this tendency, and at the same time to avoid seeing only what they want to see in the data. For example, some were challenged to see that sex workers are not always the most exploited groups of workers, and were challenged to consider that some sex workers enjoy their work, even though many feminists argue all sex workers are exploited [95,96]. At the same time, the results point to the complex interrelationships among gender, equity, and dignity in work in frontline service organizations and the need for an intersectionality perspective to help unpack this complexity.

### Reid and the Coalition for Women's Economic Advancement: Women's Employability and Health Project

Colleen Reid, a postdoctoral researcher, in collaboration with the Coalition for Women's Economic Advancement (CWEA), conducted the *Women's Employability and Health Project*. Research questions included the following: (1) How do women make a living in formal and informal economies? (2) What is the relationship between "employability" (as defined and experienced by the women themselves) and women's health and well-being? In choosing the research communities, Reid and her colleagues considered the gap between women's and men's incomes, the sources of income in the community, and the basis for the local economies. The four research communities were characterized as Rural-Farming, Northern-Resource, Remote-Reserve, and Urban-South Asian.***

The project was managed by Reid and a Project Coordinator. Local researchers were hired in each community - - in one instance, an individual, and in three others, as teams of two to four women. The community researchers were responsible for forming and liaising with a local advisory committee, recruiting participants, conducting data collection and analysis, and writing a final community report. The first contract deliverable for the local researchers was a community report to provide in-depth contextual information and relevant data on each research community. From this community snapshot they then recruited a broadly representative range of research participants; that is, local women who identified with the background information guiding the project (i.e., the erosion of the social safety net, women's experiences of barriers in finding work, being underpaid and working in the informal economy - e.g., under the table, sex trade, drug trade, and so on. In total, 60 women from across BC participated.

Qualitative methods were used including semi-structured, one-on-one interviews, and focus groups. Data analysis was conducted entirely in the local communities by the local researchers. Reid provided a descriptive coding framework, which was used by the community researchers for coding and analysis, as well as ongoing training and support. Atlas.ti was used to manage and sort the data. From the descriptive coding, the local researchers wrote a community report that integrated the contextual information from their communities with the research findings.****

From the outset, the co-researchers' intention was to connect women's lived experiences to broader systemic inequalities such as sexism and racism using an intersectionality analysis. For example, they hoped to locate women's caregiving roles and the decisions and compromises that result from adopting that role, within neo-conservative economic and social policies that have stripped provincial and community supports for unpaid caregiving. Further, the researchers hoped to generate evidence that broadened their thinking about caregiving roles such that they could better understand how characteristics of local economy, geography, and culture had similar and different impacts on women's experiences of caregiving.

What emerged as the most difficult aspect of conducting the research was a tension between the project leaders' overt feminist and intersectionality goals and the community researchers' sensitivity to the local politics and perspectives of their communities. While Reid and the CWEA were interested in analyzing the impact of systems of domination and oppression such as racism, sexism, and colonialism, the discussion of these broader systemic factors felt untrue to some of the community researchers who argued that their research participants would not agree with this analysis. In some of the communities, the research participants had individualistic rather than structural analyses of their own lived experiences and spoke of being survivors, personal resilience, and making do. In other communities the local analyses were embedded in notions of "community" that valued traditional unpaid work and were critical of consumerism and the resource extraction economy.

Alongside discourses of individual perseverance and the value of community were data pointing to the importance of power relations, social inequities, and complex interrelationships among gender, culture, poverty, race, and geography. Participants reported a significant and unchanging wage gap between men's and women's work, experiences of sexism and racism in the workplace, past and current experiences of violence and harassment, unrelenting experiences of deprivation and poverty, powerful cultural expectations for women to care for children and ailing parents, and chronic stress, depression and worry as a result of one's living conditions.

In conducting an intersectionality analysis, Reid and her colleagues uncovered how layered an intersectionality analysis can be. Not only did it raise important questions about valuing diverse and competing analyses, but it also uncovered Reid's assumptions in structuring the project itself. The project was structured with local researchers hired in each research community to conduct the research. While it made sense to have the research conducted by someone who lived in the local research community, inherent in this decision was the assumption that this person was capable of defining her community and representing the diverse perspectives of her community. As Reid and the community researchers embarked on collaborative analysis and writing, they found that the application of an intersectionality analysis to both the research findings and to the research team itself illuminated the layered complexities within the project.

These tensions raised important questions about conducting intersectionality analyses: Why is an intersectionality analysis important? What is the social justice issue being examined? Is there agreement on the core issue? Who is the research for? Who benefits and who is potentially at risk in conducting an intersectionality analysis? Are the benefits and risks borne equally? How does the "community" benefit from an intersectionality analysis? How does one bridge theoretical discussions of intersectionality with one's community? These questions forced the research team to confront the differing statuses on the research team itself. Ultimately, the team had to examine the power and impact of research and the reasons for engaging in the project in the first place. In moving forward, members of the research team agreed that the discussions about engaging in an intersectionality analysis deepened the analysis produced, raised its complexities while forcing the development of strategies to understand the complexities, and kept the analysis grounded in the research participants' lived experiences.

## Discussion

While the promise of intersectionality for creating new approaches to women's health research, and health research more broadly, has been widely acknowledged[35,49,97,98], intersectionality frameworks remain under-utilized because limited progress has been made in developing theoretically informed and methodologically sound approaches for their health research application. The five examples of Canadian research highlighted above represent work which is grounded within an intersectionality framework. They do not represent a unified way to conduct intersectionality research, but instead were chosen to demonstrate the different ways in which an intersectionality perspective can be applied to women's health research. In particular, each research team interpreted and incorporated the basic assumptions of intersectionality in their own unique way.

One of the defining characteristics of an intersectionality approach, and one which is a common theme in the above research, is that it involves the creation of coalitions and strategic alliances to alleviate poverty, social exclusion, marginalization, and subordination. Research teams were engaged in projects that were *for*, rather than about, women[54,99,100] and each strived to insure the meaningful participation of diverse women (and in some cases, men) within each community. While united in pursuing this purpose, research teams adopted a different means of achieving this goal. Brotman et al., Varcoe & Dick, Clark & Hunt, and Benoit & colleagues created a research team which involved the meaningful participation of all key stakeholder groups in all phases of the research including the identification of research questions, the design and undertaking of the research, and the dissemination of the research findings. Reid and colleagues applied a community-based research model whereby community researchers were hired as representatives of their communities.

The need for diverse participants called for by an intersectionality approach led to decisions that may be considered unconventional, or worse, undermining in women's health research. For example, the projects by Benoit & colleagues, Brotman & colleagues, and Varcoe & Dick engaged men as participants. Building linkages and collaborations across gender categories, while at the same time providing space for women's issues to be articulated by women themselves, is essential to ensuring that women's health issues are not marginalized when working on research that incorporates the perspectives of both men and women. In Brotman et al.'s research, a gender-equity stance is explicitly named to call attention to the historic invisibility of lesbian perspectives in much of health research generally and queer health research specifically and to identify ways in which lesbian realities could be named, examined, and responded to in all aspects of study design and implementation.

With its focus on partnership, participation, and reflexivity, a community-based research approach can be a useful tool for advancing intersectionality methodologies. While Reid and the Coalition experienced tensions among members of the research team who had disparate views on the importance of applying an intersectionality analysis in their study, their attempts at considering the multiple layers of intersectionality (from the data to the configuration of the research team itself), produced an enhanced and more sophisticated analysis. Varcoe and Dick's approach of including broader representation of key stakeholders in the research team and using a bottom-up approach to designing and conducting the research proved to be a success, but the loss of interest from some of the stakeholder groups over the life of the project may have long term implications for the community that participated in the research. Similarly, Brotman and colleagues' and Benoit and colleagues' decision to engage with key groups advocating on behalf of the research participants ensured the meaningful translation of research findings into appropriate programs and services from which the participants themselves would benefit.

Clark and Hunt spent a considerable amount of time, energy, and thought into developing "Intersectional Research Teams" (IRT), an approach whereby research team members were encouraged to think about their own intersectional identities, how they relate to other team members, and how their own perspectives deeply shape all aspects of the research process. As argued by Clark and Hunt, intersectionality is not only a theoretical construct to be applied to study findings. The development and practice of IRTs enables greater insight into relationships between insider and outsider and the spaces in between. The concept of IRTs shows promise for addressing the importance and challenge of involving others, such as community partners, in research; to keep the research honest, relevant, and representative; and to ensure that the analyses are grounded in the lived experiences of the participants.

Ideally, researchers using an intersectionality framework intend to place the importance of power in the research process front and centre to ensure that efforts to better understand the rich complexity and diversity of women's health experiences and needs "do not inadvertently disadvantage or harm any particular individual or community, or alternatively be complicit in the empowerment of another"[101] (p. 4). The importance of power in creating and perpetuating personal and social structures of discrimination and oppression extends directly into the realm of women's health/gender and health research. Even the most well-intentioned researchers often fail to recognize the extent to which any research project is a *process *of social construction in which discourses about categories and differences are both produced and reproduced in a way that often causes harm and undermines autonomy and empowerment of those who are vulnerable and marginalized.

Each research team explicitly analyzed the impact of broader systems of discrimination and oppression, (that is, heterosexism, colonialism, sexism, classism, and racism) on participants' lived experiences. For example, in the case of Brotman and her colleagues, the authors found that older lesbian women reported feeling marginalized by the women's health and seniors' movements which ignore sexual orientation-based oppression; and by the gay health movement which ignores gender-based oppression; and, which ultimately resulted in poorer health outcomes for this group in particular.

Unique and unexpected challenges arose that raised questions regarding whether the use of an intersectionality analysis to explore systems of power was problematic or in the case of Reid and colleagues, unwelcome. Varcoe reported that drawing attention to the role of colonialism and racism in Aboriginal women's lives may serve to inadvertently reinforce negative stereotypes. The challenge for her research team was to recognize their own assumptions and to resist essentializing the experiences of Aboriginal women (that is, assuming all Aboriginal women have similar lived experience). Brotman and colleagues also found themselves challenged by the tensions that exist in exploring oppression (which can lead to an over-emphasis on exposing only aspects of victimization among gay and lesbian seniors) and ensuring that agency, resilience, and resistance to domination are equally identified. This proved most problematic in knowledge exchange encounters with mainstream health care agencies and the media who were often more interested in the portrayal of gay and lesbian seniors as lonely, fearful, and isolated from community rather than as diversely situated individuals who can be both isolated and connected, very private about their sexual orientation, or openly engaged as activists depending upon individual experience and intersecting social locations. Benoit and colleagues also admitted surprise in finding that, prior to conducting intersectionality analyses, there was a tendency to slip into essentializing the experiences of sex workers, for example. While Reid and her colleagues intended to explore the impact of broad shifts in economic and social policy on the lives of women, this type of intersectionality analysis was challenged by the community researchers. This raises the question of *Who is the research for and does it advance the needs of those under study?*

While the concept of power within the research process was an important element of all five research projects, confronting power relationships among researchers, participants, and collaborators was also an important consideration. Weber has observed: "Incorporating a power analysis is challenging in part because knowledge about power dynamics and the processes of change has come largely when researchers have consciously chosen to confront power hierarchies in specific social contexts....including those hierarchies of power between researchers themselves and the communities they study"[53] (p. 45). Confronting and relinquishing power, becoming vulnerable, and "feeling threatened to the core"[102] are not things which even the most well-intentioned researchers are always prepared to do.

Varcoe presents a good example of the need for researchers to explore their position within the research with the recognition that her own unique social location contributed to her assumptions about the impact of colonization on Aboriginal women's lives, as well as her tendency to essentialize the experiences of Aboriginal women and rural women. Brotman and her colleagues challenged and expanded upon the queer health movement historically led by a group of relatively privileged queer people and which has largely focused on the perspective of white, middle-class, young, able-bodied, urban males. Tensions arose between Reid and colleagues and the community-based researchers involved in her study when the desire to conduct feminist and intersectionality analyses proved to be in conflict with the agenda and perspectives of the community under study. Through their creation of Intersectional Research Teams, Clark and Hunt attempted to address these issues prior to designing and conducting a research project. Members of Intersectional Research Teams were encouraged to explore and identify their own social location and the relationship their identity has to the research being conducted.

Cole[20] further suggests the following questions to fully explore the relationship between the researcher and the researched and its impact on the overall design of the research: How can coalitions among members of social groups with unequal political and economic power avoid reproducing existing inequality in their practice? What procedures will safeguard the voices and interests of the less powerful? How will agendas be set? How will human commonalities and differences be recognized without resorting to essentialism, false universalism, or obliviousness to historical and contemporary patterns of inequality? By addressing these concerns, collaborative intersectionality research teams can be seen as coalitions who "join forces across difference and requires resisting the impulse to seclude ourselves with similar others"[20] (p. 443-444), and which further, help "people move beyond their comfort zones to face, understand and accept difference" (p. 444).

## Conclusions

In the Canadian context, it has been recognized that there is a need for "targeted research with and on diverse groups"[13] so that the full range of vulnerabilities, experiences, and issues of diverse women are not obscured. A promising direction for this work is intersectionality - a normative and empirical research paradigm for studying, understanding, and responding to the ways in which gender intersects with other identities, contributing to unique experiences of oppression and privilege. Intersectionality has the potential to transform mainstream women's health research and policy. At the most general level, as Weber[53] argues, intersectionality is concerned with "building broader understandings of social inequality and health disparities" (p. 35). It provides important insights into why a primary focus on any axis of discrimination, (such as sex, gender, race or class for example), obscures the significance of other factors. The lack of attention to some differences over others also produces analyses that are less analytically sound than would otherwise be the case[103]. Additionally, such research fails to capture the underlying causes of many marginalized women's oppressions and underlying causes of poor health. Intersectionality therefore directs attention to how claims about women's health often produce hegemonic generalizations "in that they represent the problems of privileged women (most often white, Western, middle-class, heterosexual women) as paradigmatic 'women's issues'."[104] (p. 80). In turn, as a minimum, this perspective has the potential to broaden the field of women's health research to explicitly address health conditions that are more serious for women and in particular, certain groups of women. It may also, however, challenge the utility of examining health experiences within the category or field of women' health and instead to move towards fields of examination that capture the true complexities of people's lives that include but are not limited to gender.

By highlighting a few key Canadian developments, this paper reveals the usefulness of an intersectionality approach for furthering the women's health agenda. The featured projects and questions they raise are united in that they all are explicitly attentive to power and social locations in terms of researchers, research processes, and outcomes. In ascribing meaning and interpreting difference in their respective projects, all the researchers strive to produce research findings that inform policies and practices and that are intended to benefit the diversity of Canadian women, helping them more effectively meet their health care needs.

In order for the full potential of intersectionality to be realized in women's health research, however, more methodological development is needed so that research design can reflect innovative thinking about identity, equity, and power. Through exposing some of the methodological realities of engaging with an intersectional framework, this paper aimed to elucidate the many promises of intersectionality. Yet this work is in its infancy - - intersectionality demands that researchers understand how, they themselves and people living and working in community, live at multiple, fluid, and ever-changing intersections. Researchers have a resounding impact on how research is done. Further, when research is based on participatory and action-oriented values and principles, attention to an intersectionality framework demands self-examination and reflexivity. Indeed, this will require building on the theoretical and methodological innovations to date and the further development of research approaches that cross and combine disciplines and draw on novel uses and mixes of methodologies and methods. Building on some of these promising Canadian examples, more work is also required to develop effective ways to use quantitative, qualitative, and/or mixed methods to transcend singular categories of social identity such as gender, so that the consequences of the complex combination of various oppressions is captured, and in the process, power, privilege, and intersecting domains of inclusion, exclusion, and health inequalities are better comprehended and addressed.

## Appendix

### Notes

*) These examples were first presented and/or discussed and analyzed at the first Canadian conference on intersectionality research entitled "Intersectionality from Theory to Practice: An Interdisciplinary Dialogue," Institute for Critical Studies in Gender and Health, Simon Fraser University and the Women's Health Research Network, Vancouver, Canada, April 17-18, 2008.

**) There is a parallel study occurring in Sacramento, California, where the three occupational groups face much more formidable challenges accessing primary and other health and social services. The data from the two studies are being combined at this time of writing for future analyses.

***) In our ethical agreement with the Vancouver Foundation we agreed to maintain community confidentiality for the purposes of academic dissemination (e.g. journal article and book chapter).

****) These reports can be found at: http://thesurvey.womenshealthdata.ca/findaresource/Default.aspx

## Competing interests

The authors declare that they have no competing interests.

## Authors' contributions

OH, CR, and RC were equally responsible for drafting of the intellectual content of the manuscript. CR, CB, SB, NC, and CV were each responsible for describing their work under the *Canadian Examples of Intersectional-Type Research *section and also provided critical feedback on the whole manuscript. All authors approved the final manuscript.
